# A Microfluidic Platform for Investigating Transmembrane Pressure-Induced Glomerular Leakage

**DOI:** 10.3390/mi9050228

**Published:** 2018-05-10

**Authors:** Ting-Hsuan Chen, Jie-Sheng Chen, Yi-Ching Ko, Jyun-Wei Chen, Hsueh-Yao Chu, Chih-Shuan Lu, Chiao-Wen Chu, Hsiang-Hao Hsu, Fan-Gang Tseng

**Affiliations:** 1 Department of Mechanical and Biomedical Engineering, City University of Hong Kong, Hong Kong, China; thchen@cityu.edu.hk; 2 Department of Engineering and System Science, Frontier Research Center on Fundamental and Applied Sciences of Matters, National Tsing Hua University, Hsinchu 30013, Taiwan; yochenjason@gmail.com (J.-S.C.); nice9763n0293@yahoo.com.tw (J.-W.C.); kecokoyo@gmail.com (H.-Y.C.); aanny80715@livemail.tw (C.-W.C.); 3 Department of Nephrology, Kidney Research Center, Chang Gung Memorial Hospital, Chang Gung University, College of Medicine, Taoyuan 33305, Taiwan; koyc0429@gmail.com; 4 Institute of Nano Engineering and Microsystems, National Tsing Hua University, Hsinchu 30013, Taiwan; c98998@gmail.com

**Keywords:** podocytes, glomerular leakage, transmembrane pressure, microfluidics

## Abstract

Transmembrane pressure across the glomerular filter barrier may underlie renal failure. However, studies of renal failure have been difficult owing to a lack of in vitro models to capture the transmembrane pressure in a controlled approach. Here we report a microfluidic platform of podocyte culture to investigate transmembrane pressure induced glomerular leakage. Podocytes, the glomerular epithelial cells essential for filtration function, were cultivated on a porous membrane supplied with transmembrane pressure Δ*P*. An anodic aluminum oxide membrane with collagen coating was used as the porous membrane, and the filtration function was evaluated using dextrans of different sizes. The results show that dextran in 20 kDa and 70 kDa can penetrate the podocyte membrane, whereas dextran in 500 kDa was blocked until Δ*P* ≥ 60 mmHg, which resembles the filtration function when Δ*P* was in the range of a healthy kidney (Δ*P* < 60 mmHg) as well as the hypertension-induced glomerular leakage (Δ*P* ≥ 60 mmHg). Additionally, analysis showed that synaptopodin and actin were also downregulated when Δ*P* > 30 mmHg, indicating that the dysfunction of renal filtration is correlated with the reduction of synaptopodin expression and disorganized actin cytoskeleton. Taking together, our microfluidic platform enables the investigation of transmembrane pressure in glomerular filter membrane, with potential implications for drug development in the future.

## 1. Introduction

Transmembrane pressure across the basement membrane that separates epithelium and vascular endothelium may underlie the transportation of fluids, nutrients, or other metabolic factors across tissue interfaces and affect neighboring parenchymal tissues. In particular, it has an important role in glomeruli, the basic units of the kidney’s filtration function. Inside a glomerulus, the glomerular filter barrier consists of three cell layers [1]. Among them, the podocyte is a highly specialized cell type responsible for the glomerular permselectivity of proteins in the kidney [2]. Differentiated podocytes are mesenchymal-like cells with foot processes, a highly branched inter-digitating network. In between foot processes of opposing podocytes, the filtration slits establish the final barrier to urinary protein loss [3]. Thus, the factors that change the podocyte phenotype have attracted considerable attention as small changes of actin cytoskeleton may result in disappearance of actin-enriched foot processes and damage the filtration function [4,5,6], eventually causing leakage of blood cells or serum proteins into the urine [7,8]. Recent results suggest that net filtration pressure or transmembrane pressure may govern the fluidic movement across glomerular basement membrane essential for the filtration [9]. In normal conditions, the net filtration pressure is 10–30 mmHg [9]. However, it may exceed 60 mmHg in the condition of hypertension [10]. Thus, the pronounced increase of trans-glomerular pressure gradient that results in the enhanced mechanical stress may be an important cause of podocyte injury [11,12] and can eventually disrupt the renal filtration barrier [13].

There are many mechanical platforms for studying mechanical factors on cell activities. For example, microfluidics has been used to study shear stress in endothelial cell alignment and adhesion [14,15,16]. Mechanical compression or stretching was also applied to the entire culture [17,18,19] for studying the force dependence of cell orientation. In particular, because cells are pre-stressed with internal contractile forces, attachment to a more compliable substrate would cause a reduction in cell size as well as the internal forces [20,21,22], which later results in faster migration and optimized proliferation [22]. However, the existing platforms lack the ability to recapture the transmembrane pressure for cultured cells in a controllable manner [23], creating a difficulty when investigating the role of transmembrane pressure in physiology/pathology of glomerulus.

**Figure 1:**
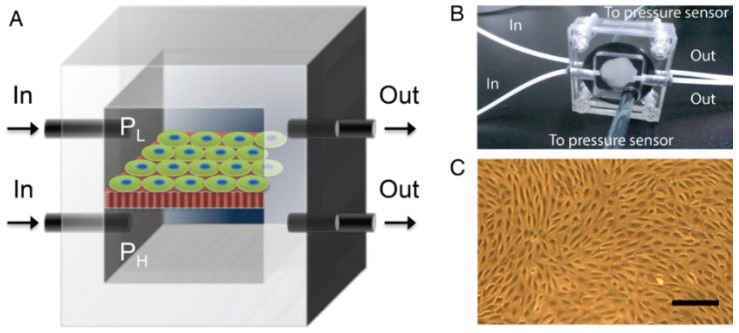
Overview of the microfluidic platform. (**A**) Schematics of the device where podocytes were cultured on porous membrane, which was sandwiched by an upper chamber supplying *P*_L_ and a lower chamber supplying with *P*_H_. The pressures in both chambers were controlled by peristaltic pumps that infused the medium via the inlet and outlet and monitored by pressure sensors. (**B**) An image of the real device; (**C**) a microscopic image of the differentiated podocytes monolayer. Scale bar, 50 μm.

Here we demonstrate a microfluidics-based in vitro model to investigate transmembrane pressure induced glomerular leakage. The schematic is shown in Figure 1. The glomerular filtration membrane was represented by a monolayer of podocytes with 100% confluence. The podocyte monolayer was cultured on an anodic aluminum oxide (AAO) membrane consisting of nanoscale transmembrane pores [11]. The membrane was sandwiched between two acrylic chambers to provide the transmembrane pressure (Δ*P*), i.e., the net pressure difference between *P*_H_ in the lower chamber to simulate glomerular capillaries hydrostatic pressure and *P*_L_ in the upper chamber to simulate the combination of colloid osmotic pressure and Bowman’s hydrostatic pressure. A collagen-coated anodic aluminum oxide membrane was used to simulate the glomerular basement membrane. The filtration function was validated using dextran with different sizes. We found that dextran in 20 kDa and 70 kDa can penetrate the podocyte membrane whereas dextran in 500 kDa was blocked until Δ*P* ≥ 60 mmHg, which resembles the filtration function when Δ*P* was in the range of healthy kidney (Δ*P* < 60 mmHg) as well as the hypertension-induced glomerular leakage (Δ*P* ≥ 60 mmHg). Also, such damage was not self-repairable even if 10 mmHg of Δ*P* was subsequently applied. With the evidences showing downregulated expression of synaptopodin and disorganized actin cytoskeleton in high Δ*P*, our model suggests that high transmembrane pressure may result in a change of podocyte phenotype, eventually disrupting the filtration function. As an in vitro model recapturing transmembrane pressure in physiology/pathology of glomerulus, this approach paves the way for future drug development and potential therapy for pressure-induced renal failure.

## 2. Materials and Methods

### 2.1. Podocyte Culture

We used conditionally immortalized cells with stable expression of nephrin and formation of cell–cell contacts derived by Endlich’s group [24]. To cultivate them, in brief, podocytes were transfected with a temperature-sensitive T antigen such that they become conditionally immortalized at 33 °C. The growth medium consists of RPMI 1640 (Corning, Corning, VA, USA) and 10% fetal bovine serum (Corning, Corning, VA, USA) supplemented with 20–100 U/mL of mouse interferon gamma (INF-γ, Miltenyi Biotec, Bergisch Gladbach, Germany) to drive T-antigen expression. Later, to inactivate the T antigen that exits the cell proliferation cycle and promotes differentiation, the cultures were switched to medium lacking INF and transferred to an incubator set at 37 °C [25].

### 2.2. Podocyte Spreading and Proliferation on Porous Membrane

To resemble the filtration barrier, porous membranes were used in the podocyte culture to mimic the glomerular basement membrane. The AAO membranes (Whatman, Inc., Little Chalfont, UK) with pore size 0.02 μm or 0.2 μm were used to culture the podocytes. For comparison, we also used polycarbonate (PC) membranes with 0.5 μm and 5 μm pore size (Whatman, Inc., Little Chalfont, UK) with lower porosity.

Before plating the podocytes, the membrane was sterilized in 95% alcohol for 10 min and exposed to UV for 24 h. The membranes were either directly plated with cells or pre-coated with type I collagen (BD Biosciences, San Jose, CA, USA) in 0.8 mg/mL solution for 24 h before cell seeding. The podocytes were plated on the membranes at a density of 10,000 cells/cm^2^ and cultured in a six-well culture dish. To study the cell proliferation, the cultures were conducted at 33 °C for a total of five days. For studying the cell spreading, the culture was started at 33 °C with regular growth medium for 48 h and then changed to 37 °C for another 48 h using culture medium lacking INF-γ. The culture medium was changed every day. At the time of measurement, the proliferation was obtained by counting the increase in cell numbers. For assessing the cell spreading, live cells were stained by 0.1% Calcein AM (Life Technology, Carlsbad, CA, USA) in PBS at 37 °C for 15 min to observe the morphology in the following days using a fluorescence microscope.

### 2.3. Pressure-Supplying Device

The membrane was sandwiched between upper and lower acrylic chambers separately supplied with fresh medium (Figure 1). The pressure difference was achieved by peristaltic pumps and monitored by pressure sensors. Before attaching to the chambers, the membrane was coated with collagen for 30 min. Next, podocytes with 10,000 cells/cm^2^ were first cultured at 33 °C for three days to reach 60% confluence, followed by eight days in 37 °C for differentiation. Next, the membrane was sandwiched by the acrylic chambers and placed into an on-stage incubator to keep the culture under 37 °C and 5% CO_2_ infusion. The upper chamber was supplied with lower pressure (*P*_L_), which was adjusted for different Δ*P*, while the lower chamber was supplied with higher pressure (*P*_H_) and kept as a constant. Note that podocytes were facing the chamber with lower pressure to simulate in vivo circumstances.

### 2.4. Dextran Filtration

The Δ*P* was applied to the AAO membrane with confluent podocytes for 2 h. To evaluate the filtration function with different Δ*P*, the medium in both the upper and lower chambers was first removed, followed by infusing the medium with dextran into the lower chamber only. Dextrans with three representative sizes were used: 20 kDa, 70 kDa, and 500 kDa (Nanocs Inc., New York, NY, USA). One minute after infusion, the medium that penetrated from the lower chamber to the upper chamber was collected. To evaluate the self-repairing, 70 mmHg of Δ*P* was applied for 6 h, followed by supplying lower Δ*P* (10 mmHg) for another 6 h. Dextrans penetration samples were collected at 2 h, 6 h, 8 h and 12 h. The amount of dextran was measured using its fluorescent intensity by a plate reader (Victor^3^, Perkin Elmer, Waltham, MA, USA).

### 2.5. Quantitative Real-Time Polymerase Chain Reaction (q-PCR)

Total RNA was isolated using TRIzol reagent (Life Technology) according to the manufacturer’s instructions. q-PCR was performed on ViiA™ 7 Real-Time PCR System (Applied Biosystems, Foster City, CA, USA). The gene expression was analyzed using TaqMan^®^ Gene Expression Assay (Life Technology) containing FAM dye-labeled probe and primers (Assay ID: GAPDH, Mm99999915_g1; 18 S, Hs99999901_s1; αActin-4, Mm00502489_m1, and Synaptopodin, Mm03413333_m1) following manufacturer’s instructions. The comparative ∆C_T_ method was used for relative quantification of mRNA expression. 18S were used as endogenous controls to obtain the ∆*C*_T_, and wild-type podocytes cultured in 37 °C without Δ*P* were used as the calibrator to obtain the ∆∆*C*_T_. Finally, the relative mRNA expression was quantified by 2^−∆∆*C*T^ as the final readout.

### 2.6. Fluorescence Staining of Actin

After application of Δ*P*, the cells were fixed in 4% formaldehyde at 4 °C for 15 min, followed by PBS rinsing three times. Next, 1% BSA in PBS was applied for 30 min for blocking, followed by incubation of phalloidin (1:100, Sigma-Aldrich, St. Louis, MO, USA) for 1 h and rinsing by PBS before image acquisition.

## 3. Results

### 3.1. Cell Proliferation on Porous Membranes

We first studied the podocyte proliferation when cultured on porous membrane. Podocytes have been the center of focus because of the formation of foot processing and slit diaphragms, which are known as a final barrier to prevent urinary protein loss [26,27,28]. More importantly, unlike vascular endothelial cells, podocytes do not regenerate, suggesting a closer relationship to the progressive loss of renal filtration function observed in chronic kidney disease. Thus, while microvascular endothelial cells may also play a role in glomerular filtration and participate in the progression of glomerular disease [29], podocytes were the focus here in order to investigate the self-perpetuation of renal failure.

**Figure 2:**
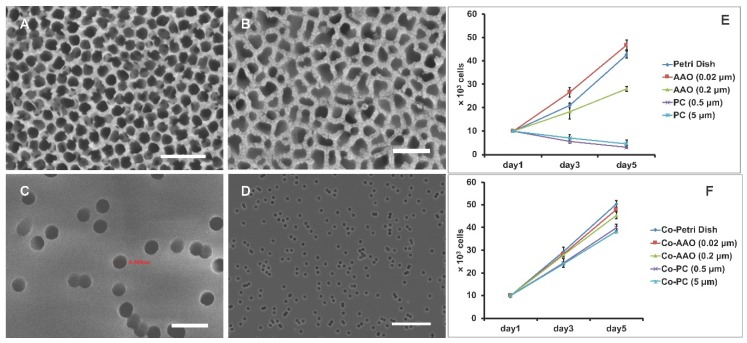
SEM images for AAO membrane with (**A**) 0.2 μm and (**B**) 0.02 μm pores, and PC membrane with (**C**) 0.5 μm and (**D**) 5 μm pores. Scale bar, 1 μm (**A**,**C**), 100 nm (**B**), and 50 μm (**D**). (**E**) The growth curve of podocytes on AAO or PC membrane without collagen coating. (**F**) The growth curve of podocytes on AAO or PC membrane with collagen coating. For (**E**,**F**), data are shown as mean ± SD (*n* = 3).

Podocytes cultured on an AAO membrane were compared with those on PC membrane with lower porosity and regular petri dish with zero porosity. Scanning electronic microscopy (SEM) showed that the AAO membrane is compo and kept as a constant sed of nanoscale pores with a density as high as 50%, providing a topology closer to that of the glomerular basement membrane. Also, the actual pore size of AAO membranes 0.02 μm is in fact much larger than 0.02 µm (Figure 2A–D), which allows for the penetration of a big dextran fraction of 500 kDa, which has a Stoke’s radius of 10–15 nm.

The results of cell proliferation showed that, on an AAO membrane and on a petri dish without any coating, the cell number doubled in about 48 h at 33 °C, and they grew faster on 0.02 μm pores than on 0.2 μm pores or on a petri dish. By contrast, on a PC membrane, increased cell death was observed regardless of the PC membrane’s pore size (Figure 2E). Thus, the results indicate that podocytes prefer to grow on an AAO membrane or on a petri dish than on a PC membrane. Also, smaller pores on the AAO membrane provide an additional positive effect for cell proliferation.

Next, we coated collagen on the same sets of membranes for 24 h prior to plating cells. As the topology remained, the material difference between the AAO, petri dish, and PC membrane was overridden by the coverage of collagen. The results showed that the cell number increased for all cases (Figure 2F). Of note, on a PC membrane with a collagen coating, the growth rate rose to a level similar to that on AAO membranes, suggesting that the lower proliferation on a PC membrane without coating is due to the material difference but not the topology.

### 3.2. Cell Spreading on Porous Membranes

**Figure 3:**
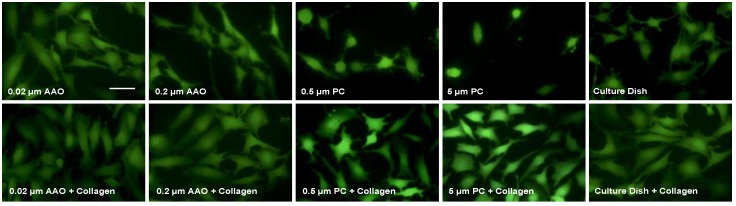
Podocytes incubated at 33 °C for 48 h. The images were taken by fluorescence microscopy. Scale bar, 5 μm.

We further investigated the cell spreading in response to different membranes. Calcein staining was used to reveal the cell shapes. The results showed that, when collagen coating is absent, cells on AAO membranes spread to a higher degree than on PC membranes and on a petri dish (Figure 3, upper row). This suggests that AAO membranes can promote cell spreading, which may explain the increased proliferation before applying a collagen coating. We then tested whether the collagen coating, which was shown to increase cell proliferation, can also have a positive effect on cell spreading. After coating with collagen on AAO and a PC membrane, podocytes showed higher spreading than that on non-coated ones (Figure 3, lower row). Such an increase was also observed in cells on a petri dish.

**Figure 4:**
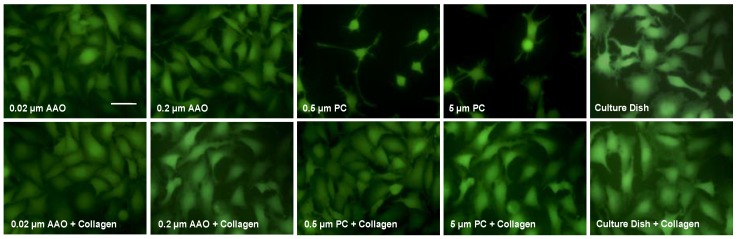
Podocytes incubated at 37 °C for 48 h. The images were taken by fluorescence microscopy. Scale bar, 5 μm.

We also cultured podocytes at 37 °C and studied their morphology (Figure 4). The effect is similar to the results from culture at 33 °C. However, compared to cells cultured at 33 °C, where cell morphology seems polygonal and plane, podocytes cultured in 37 °C spread with an arborized shape and extended more foot processes (Figure 4, upper row). When the membranes were coated with collagen, this morphology was observed on both AAO and PC membranes (Figure 4, lower row). Thus, consistent with the former results of podocyte proliferation, cell spreading was also increased by collagen coating regardless of the underneath materials.

### 3.3. Filtration Function in Response to ΔP

**Figure 5:**
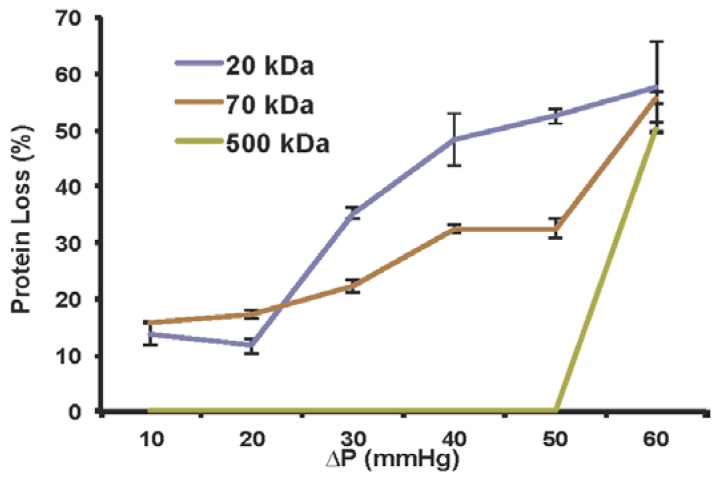
Filtration capability of podocyte membrane using dextran with sizes of 20 kDa, 70 kDa, or 500 kDa. Data are shown as mean ± SD (*n* = 3).

We next implemented the transmembrane pressure to evaluate the filtration function. We selected an AAO membrane with a 0.02 μm pore size because it is composed of nanoscale pores with a density as high as 50%, providing a topology closer to that of the glomerular basement membrane. Also, collagen coating was used as suggested by the studies of cell proliferation and spreading. Membranes with confluent and differentiated podocytes were attached to the upper and lower acrylic pressure chambers. Medium was pumped into the chambers and Δ*P* was monitored by pressure meters in each chamber (Figure 1). After supplying Δ*P* for 2 h, dextran was added to the medium to see whether a membrane with confluent podocytes can prevent transmembrane penetration. Three representative sizes of dextran were used: 20 kDa represents small molecules that usually penetrate the filtration membrane; 70 kDa represents intermediate molecules that penetrate the membrane but are reabsorbed by tubule in vivo; and 500 kDa represent big molecules that cannot penetrate the filtration membrane. At the time of measurement, the regular medium in both upper and lower chamber was removed, followed by infusing medium with dextran into the lower chamber only. One minute after infusion, the medium that penetrated into the upper chamber was collected and quantified based on its fluorescent intensity. The result showed that the penetration of dextran with size 20 kDa and 70 kDa increased when Δ*P* was increased from 10 to 60 mmHg (Figure 5). In addition, dextran with size 500 kDa was blocked when Δ*P* < 60 mmHg. This demonstrates the filtration capability of the podocyte culture resembling the normal physiological conditions. More importantly, for Δ*P* ≥ 60 mmHg, dextran with size of 500 kDa started to penetrate through the membrane, showing the malfunction of podocyte filtration mimicking the glomerular leakage. Thus, when low Δ*P* was supplied, the result demonstrates the recapitulation of filtration function by this in vitro model. When high Δ*P* was supplied, the simulated hypertension damaged the filtration function, suggesting the in vivo-like response.

**Figure 6:**
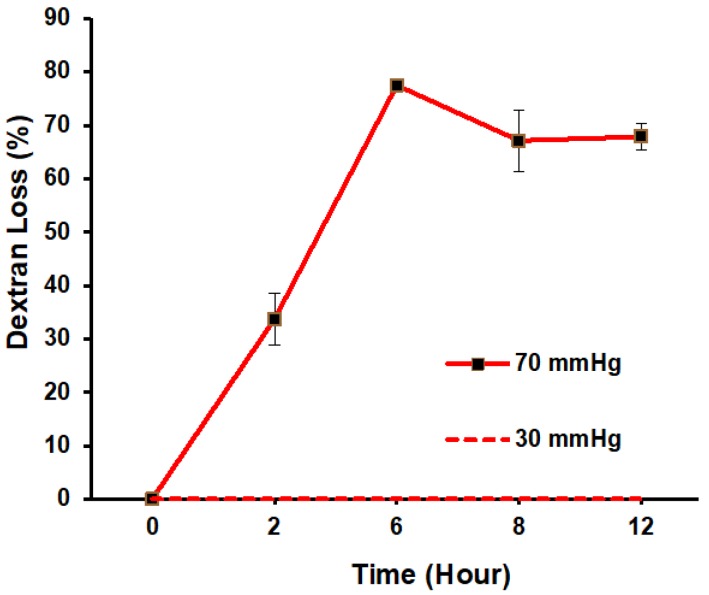
Podocyte filtration function. Δ*P* of 30 mmHg and 70 mmHg were applied for 6 h, followed by 10 mmHg for the following 6 h to investigate the recovery of filtration function. Data are shown as mean ± SD (*n* = 3).

One of the crucial features of glomerular malfunction is self-perpetuation, meaning that the condition will progress to terminal renal failure even if the initial cause is removed. Thus, we ask whether the loss of filtration function in this in vitro model is self-healable. To address this, we investigated the podocyte filtration function by applying Δ*P* at 30 mmHg or 70 mmHg for 6 h, followed by applying 10 mmHg another 6 h. Under moderate Δ*P*, 30 mmHg, results showed that there was no loss of 500 kDa dextran observed during the entire course. In contrast, by applying Δ*P* at 70 mmHg for 6 h, which is the level of hypertension, the dextran loss increased from 0% to 77.5% (Figure 6). Importantly, this damaged filtration function is not fully self-repairable. After applying Δ*P* at 10 mmHg for another 6 h, there was still 67.9% of dextran loss for 500 kDa dextran, suggesting that this in vitro model is also self-perpetuated when high Δ*P* is applied, which is similar to the pathological behavior observed in vivo.

### 3.4. Synaptopodin and Actin Expression

**Figure 7:**
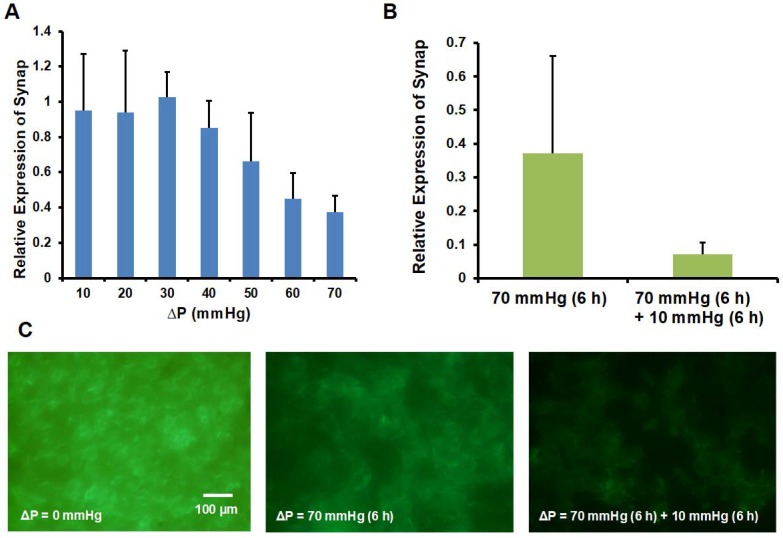
q-PCR analysis of the relative expression of (**A**) synaptopodin by applying Δ*P* for 6 h, (**B**) synaptopodin by applying Δ*P* = 70 mmHg for 6 h or Δ*P* = 70 mmHg for 6 h followed by Δ*P* = 10 mmHg for 6 h. Data are shown as mean ± SD (*n* ≥ 2). (**C**) Immunofluorescence staining of actin for culture applied with Δ*P* = 0 mmHg (left), Δ*P* = 70 mmHg for 6 h (middle) and Δ*P* = 70 mmHg for 6 h followed by Δ*P* = 10 mmHg for 6 h (right).

We further investigated whether the loss of filtration function is associated with the change of podocyte phenotype. Synaptopodin is an important marker expressed in differentiated podocytes [23]. The co-localized synaptopodin on actin filament is important for cytoskeletal rearrangement and foot processes [23,30]. Based on the mRNA expression of synaptopodin by q-PCR, we found that the expression level of synaptopodin decayed with the increase of Δ*P* (Figure 7A) and became even lower after the Δ*P* was switched to 10 mmHg for another 6 h (Figure 7B). Moreover, by staining the actin, we observed a decrease of actin expression after applying Δ*P* = 70 mmHg for 6 h and followed by Δ*P* = 10 mmHg for 6 h (Figure 7C). Combined with the results of the loss of filtration function (Figure 6), the results suggest that the non-self-healable renal failure due to high transmembrane pressure could be due to the loss of synaptopodin expression and disorganized actin cytoskeleton.

## 4. Conclusions

In summary, we established a microfluidic-based model that recapitulates glomerular leakage by transmembrane pressure. Transmembrane pressure is one of the major causes of renal failure. However, owing to the lack of in vitro models, the understanding of the disease mechanism and development of medical treatment remains limited. Based on our microfluidic platform with optimized culture condition, i.e., AAO membrane with 0.02 μm pores and collagen coating, we found that 500 kDa dextran was blocked until Δ*P* ≥ 60 mmHg, which resembles the regular filtration function and hypertension-induced glomerular leakage. Importantly, once the filtration function was damaged, this glomerular leakage was not self-repairable even after the Δ*P* was switched to 10 mmHg for a subsequent 6 h. Moreover, the synaptopodin and actin were down regulated when Δ*P* increased, suggesting that the transmembrane pressure induced glomerular leakage is closely associated with the loss of synaptopodin and actin. Together, this microfluidic device will provide a platform technology to investigate the glomerular leakage, with implications for drug development and treatment of renal failure in the future.
